# Effectiveness of information technology–enabled ‘SMART Eating’ health promotion intervention: A cluster randomized controlled trial

**DOI:** 10.1371/journal.pone.0225892

**Published:** 2020-01-10

**Authors:** Jasvir Kaur, Manmeet Kaur, Venkatesan Chakrapani, Jacqui Webster, Joseph Alvin Santos, Rajesh Kumar

**Affiliations:** 1 Department of Community Medicine and School of Public Health, Post-graduate Institute of Medical Education and Research, Chandigarh, India; 2 Centre for Sexuality and Health Research and Policy (C-SHaRP), Chennai, India; 3 The George Institute for Global Health, University of New South Wales, Sydney, Australia; 4 School of Public Health and Community Medicine, University of New South Wales, Sydney, Australia; University of Ghana, GHANA

## Abstract

**Background:**

Unhealthy dietary behaviour–high intake of fat, sugar, and salt, and low intake of fruits and vegetables–is a major risk factor for chronic diseases. There is a lack of evidence-based interventions to promote healthy dietary intake among Indian populations. Therefore, we tested the effectiveness of an information technology-enabled ‘SMART Eating’ intervention to reduce the intake of fat, sugar and salt, and to increase the intake of fruits and vegetables.

**Methods:**

In Chandigarh, a North Indian city, a cluster randomized controlled trial was implemented in twelve geographical clusters, based on the type of housing (i.e., LIG: Low-income group; MIG; Middle-income group, and HIG: High-income group–a proxy for socio-economic status). Computer-generated randomization was used to allocate clusters to intervention and comparison arms after pairing on the basis of socioeconomic status and geographical distance between clusters. The sample size was 366 families per arm (N = 732). One adult per family was randomly selected as an index case to measure the change in the outcomes. For behaviour change, a multi-channel communication approach was used, which included information technology–short message service (SMS), email, social networking app and ‘SMART Eating’ website, and interpersonal communication along with distribution of a ‘SMART Eating’ kit—kitchen calendar, dining table mat, and measuring spoons. The intervention was implemented at the family level over a period of six months. The comparison group received pamphlets on nutrition education. Outcome measurements were made at 0 and 6 months post-intervention at the individual level. Primary outcomes were changes in mean dietary intakes of fat, sugar, salt, and fruit and vegetables. Secondary outcomes included changes in body mass index (BMI), blood pressure, haemoglobin, fasting plasma glucose (FPG), and serum lipids. Mixed-effects linear regression models were used to determine the net change in the outcomes in the intervention group relative to the comparison group.

**Results:**

Participants’ mean age was 53 years, a majority were women (76%), most were married (90%) and 51% had completed a college degree. All families had mobile phones, and more than 90% of these families had access to Internet through mobile phones. The intervention group had significant net mean changes of -12.5 g/day (p<0.001), -11.4 g/day (p<0.001), -0.5 g/day (p<0.001), and +71.6 g/day (p<0.001) in the intake of fat, sugar, salt, and fruit and vegetables, respectively. Similarly, significant net changes occurred for secondary outcomes: BMI -0.25 kg/m^2^, diastolic blood pressure -2.77 mm Hg, FPG -5.7 mg/dl, and triglycerides -24.2mg/dl. The intervention had no effect on haemoglobin, systolic blood pressure, low-density lipoprotein cholesterol, or high-density lipoprotein cholesterol.

**Conclusion:**

The IT-enabled ‘SMART Eating’ intervention was found to be effective in reducing fat, sugar, and salt intake, and increasing fruit and vegetable consumption among urban adults from diverse socio-economic backgrounds.

**Trial registration:**

Clinical Trial Registry of India CTRI/2016/11/007457.

## Background

Diets have undergone a big change due to industrialization and urbanization across the globe. Dietary intake may depend more on taste, culture, and affordability rather than on official dietary recommendations [1]. Unhealthy dietary behaviour–high consumption of food that have high fat, sugar, and salt, and low consumption of fruits and vegetables–is a major risk factor for chronic diseases such as cardiovascular diseases, diabetes, and certain cancers [2]. Chronic diseases contribute to more than 71% (39.5 million) of the global disease burden [3]. More than 37% of these (15 million) deaths occur under 70 years of age; a majority (85%) of these premature deaths occur in low- and middle-income countries. Cardiovascular diseases account for the largest number of deaths, i.e., 17.6 million (45%), followed by 23% from cancers, 9% from chronic respiratory diseases and 4% from diabetes [4].

India is also having epidemics of chronic diseases [5,6]. The prevalence of hypertension among urban men and women is 31% and 26%, respectively, and that of diabetes is 22% and 19%, respectively [7]. About 5.8 million people, i.e., 1 in 4 die every year from chronic diseases before the age of 70 [8].

Given that most chronic diseases arise from or worsened by unhealthy diets, adapting healthy dietary behaviours can prevent several chronic diseases [1]. The Global Strategy on Diet, Physical Activity and Health (DPAS) emphasized the need to limit consumption of salt, saturated fats, trans fatty acids and sugars, and to increase consumption of fruits and vegetables [9]. For promoting such healthy behaviours, face-to-face individual counseling or group education approaches have been often used [10]. Although these approaches are effective, they may be expensive to scale up in resource-limited settings [11]. One solution could be to use information technology, which is increasingly used by people across different socioeconomic statuses [12,13]. mHealth has the potential to reach large numbers quickly. However, the effectiveness of information technology to improve dietary behaviours has not been explored adequately as yet in developing countries [14,15].

Most populations in India, including those from rural settings, are open to the use of m-health interventions [16,17]. To date, only one study from India has tested the effectiveness of mobile phone text messages to improve diabetes risk behaviours (high fat intake, and low fruit and vegetable intake) and reported that it was effective [17]. However, the effectiveness was evaluated based on subjective binary outcome measures (e.g. number of servings of fruits and vegetables consumed/day: 0–1, 2–4, >4; yes/no; do you consistently avoid high fat food? yes/no) rather than objective measurements; which could have overestimated the intervention effect. Although an exclusive use of mobile phone in health promotion interventions may be effective for certain diseases, for reducing complex, multifactorial risk behaviours like dietary practices, use of a single technology alone may be insufficient [18]. Hence, there is a need to develop innovative interventions using multi-channel communication approaches including information technology, human interaction and social support. Therefore, the present study was undertaken to develop, and determine the effect of community-led information technology-enabled ‘SMART (Small, Measurable and Achievable dietary changes by Reducing fat, sugar and salt consumption and Trying different fruits and vegetables) Eating’ intervention on nutrition behaviour.

## Methodology

### Ethics statement

The study protocol was approved by the Institute Ethics Committee, Postgraduate Institute of Medical Education and Research, Chandigarh (INT/IEC/2015/525; Date: 12/09/2015). Both written and verbal consents of the study participants were obtained after briefing them about the study purpose.

### Trial registration

**Fig 1:**
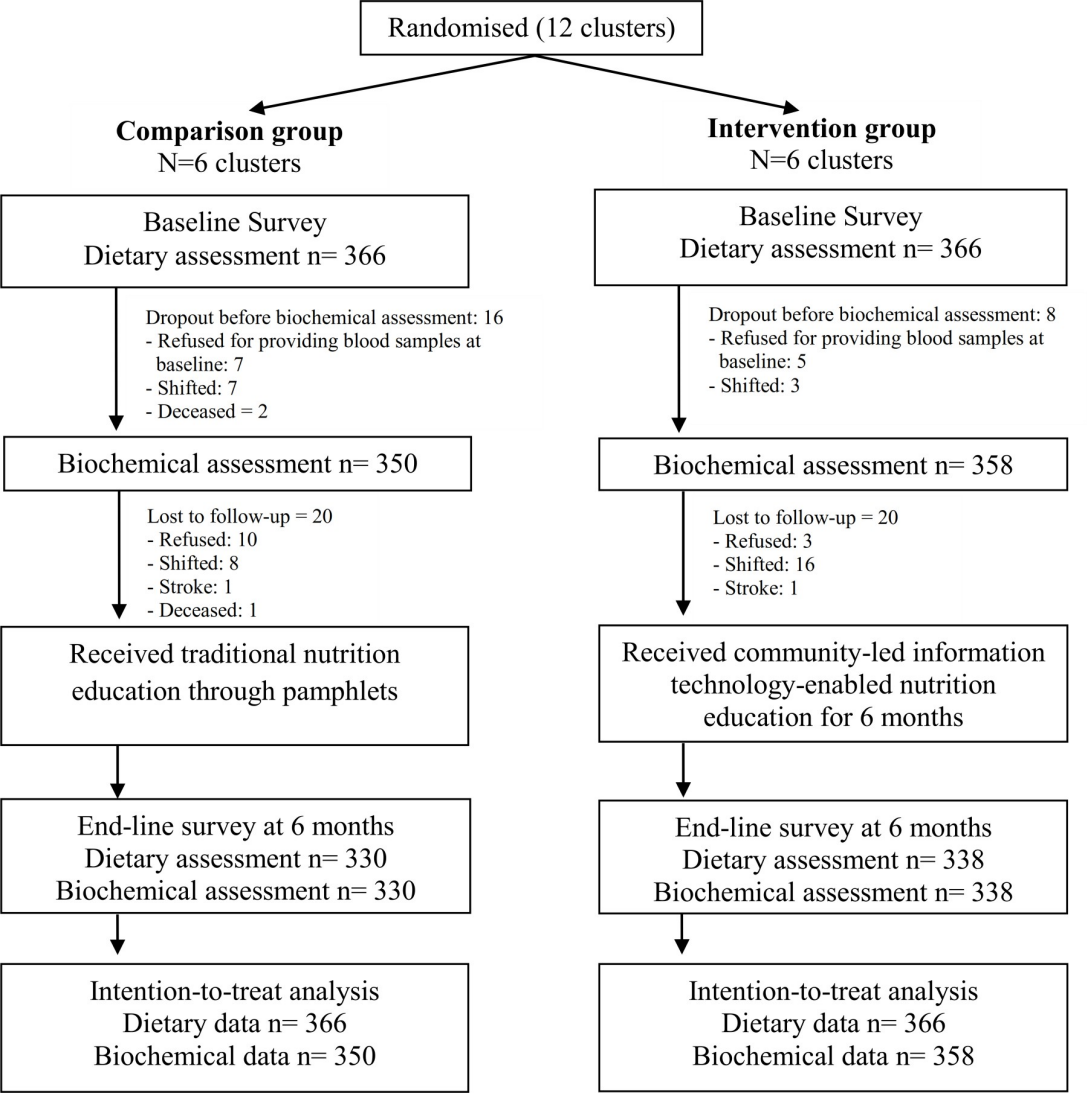
Flow of participants through the trial.

The trial details are reported in accordance with the CONSORT guidelines Fig 1 [19]. The trial was registered under the Clinical Trial Registry of India (CTRI/2016/11/007457; Date: 9/11/2016). The protocol paper with a detailed description of the study design and methodology has been published elsewhere [20].

### Study design

A two-arm cluster randomized controlled trial was conducted for testing the effectiveness of nutrition education intervention (Fig 1).

### Study setting

The study was conducted in Chandigarh city, located in northern India. Of the 63 sectors in Chandigarh city, one sector was purposively chosen for the study as our Institute has established a health centre in this sector and the use of mobile phones and internet is also widespread in this area, with more than 90% of the families having access to smart phones and internet services.

### Sampling procedure

**Fig 2:**
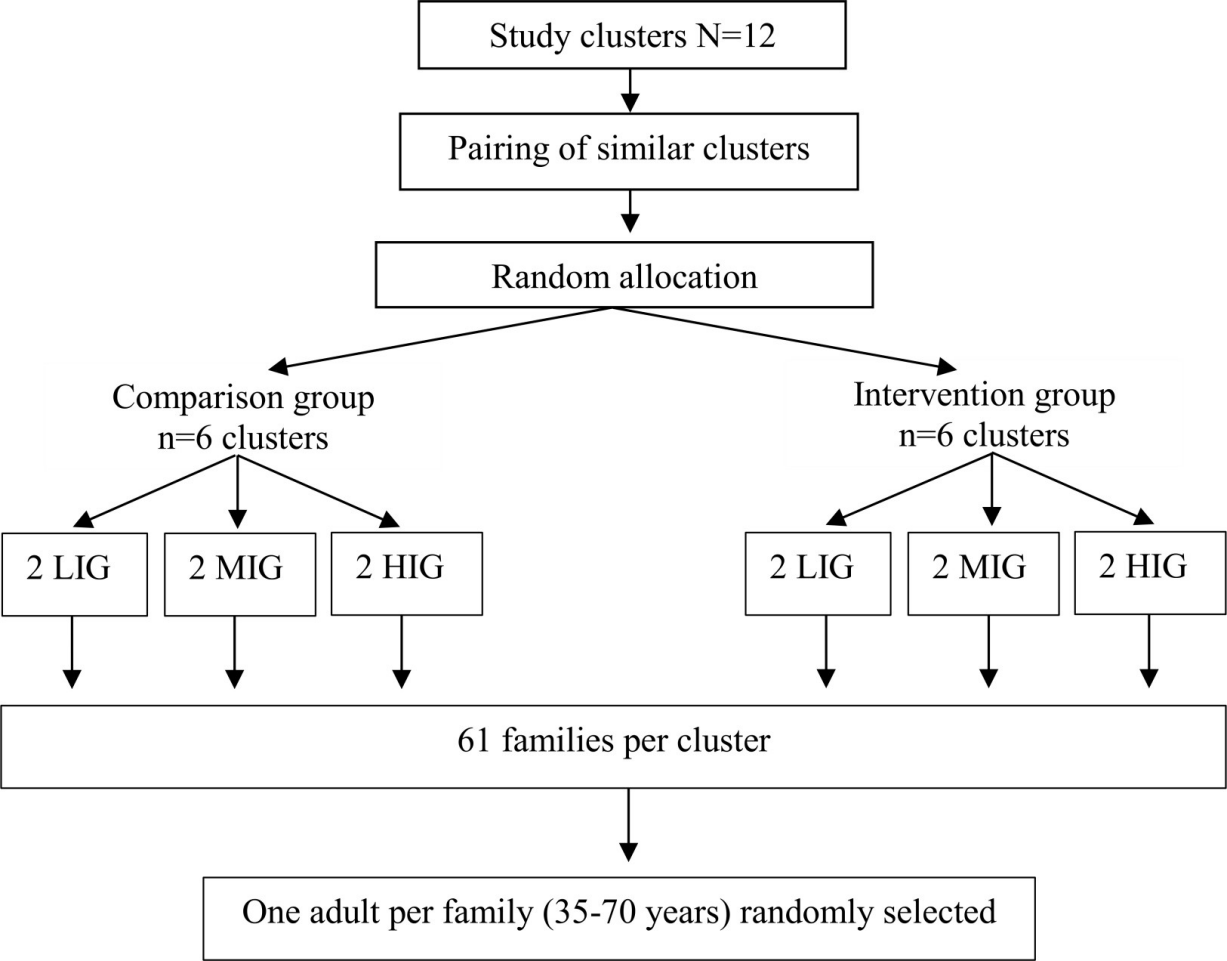
Randomisation and sampling procedure.

Type of housing, assigned by the Chandigarh Administration as low-income group (LIG), middle-income group (MIG) and high-income group (HIG), was taken as a proxy for socio-economic status. Twelve geographical clusters (4 LIGs, 4MIGs and 4 HIGs) were selected based on the type of housing (Fig 2). Within the similar socioeconomic group clusters, pairing of clusters was made on the basis of geographical distance between the clusters to form six pairs–i.e., those two clusters that were far apart within the same socioeconomic group clusters were chosen as a pair to avoid possible spill-over effect. Then, in each of the six pairs, computer-generated simple randomisation with RANDBETWEEN command in Excel 2010, was used to allocate clusters to the intervention and comparison arms with 1:1 allocation by a researcher not involved in the study. Equal numbers of families were recruited from each cluster through systematic random sampling. One adult (35–70 years) per family was randomly selected as an index case to measure the change in the outcomes of interest in the study. For intervention implementation, one family champion (an adult in the family who usually cooks food) was selected from each family. As it was revealed during the formative research that not all family champions will be using information technology tools, so one co-champion (a family member who assists family champion in using IT tools) identified by the family champion was also selected.

### Inclusion and exclusion criteria

The inclusion criteria for recruitment of study participants was families from low-, middle- and high-income group housing (LIG, MIG, HIG), residing in the study area for 6 months or more, had adults between 35 and 70 years of age, and had access to mobile phone or landline phone or Internet. Pregnant women, and families not providing consent to participate in the study were excluded.

### Sample size

Using prevalence of adequate dietary intake for the different foods and nutrients, sample sizes were calculated for each of the primary outcome variables, i.e., fat, salt, fruit and vegetable intake (except for sugar intake for which estimates were not available). Estimating change in salt intake required the largest sample size. Based on an estimated 15% prevalence of adequate dietary salt intake (<5 g/day) [21], and assuming a 20% improvement in the intervention arm compared to the comparison arm, power calculations (power = 0.80; alpha = 0.05) indicated that 83 participants would be needed per study arm. The sample size was increased to 183 to take into account a design effect of 2 [22], and a drop-out rate of 10% [23]. To allow for subgroup comparisons, the sample size was doubled (n = 366 per arm), divided equally into the 6 clusters per arm (n = 61 per cluster), which was deemed to be sufficient to assess changes in each of the primary outcomes.

### Study outcomes

Primary outcomes were changes in mean dietary intakes of fat, sugar, salt, fruit and vegetables. Secondary outcomes included changes in dietary intake of ß-carotene (μg), vitamin B-12 (μg), vitamin C (mg), folate (μg), iron (mg), sodium (mg), potassium (mg) and iodine (μg); changes in body weight, body mass index (BMI), blood pressure, haemoglobin, fasting plasma glucose (FPG), total cholesterol (TC), high-density lipoprotein cholesterol (HDL-C), low-density lipoprotein cholesterol (LDL-C), and triglycerides (TG).

### Intervention description

The intervention was guided by the PRECEDE-PROCEED model [24], empirical literature on interventions, dietary guidelines by the National Institute of Nutrition, India [25], and qualitative formative research. A detailed description of the intervention protocol has been published elsewhere [20]. Qualitative formative research, guided by the Social Ecological Model, revealed multi-level influences on dietary behaviours at individual, family, and social-structural level which facilitated the development of context-specific, culturally acceptable intervention [26]. The intervention group received IT-enabled nutrition education which had two components: 1) The interpersonal component, which included the distribution of ‘SMART Eating’ kit–kitchen calendar, dining table mat, and measuring spoons; 2) The Information Technology (IT) Component–SMS, email, social networking app, and ‘SMART Eating’ website (Table 1). The intervention was implemented at the family level through family champions over a period of six months.

**Table 1:** Description of ‘SMART Eating’ intervention components.

Intervention components	Intervention objectives	Educational aids	Duration	Implementation
**1. Interpersonal component**	• To guide family champions and co-champions on how to use different components of the intervention • To enhance self-efficacy in adapting to healthy dietary behaviours using written educational material	Flip bookProvision of ‘SMART Eating’ kit − Dining table mat − Kitchen calendar − Measuring spoons	1 month	• Single home visit was conducted. • ‘SMART Eating’ kit provided to all intervention families − A pictorial laminated dining table-mat on dietary recommendations to guide the families on seasonal availability of vegetables and fruits. The other side of the mat reminded the family members to reduce fat, sugar, and salt. − A pictorial kitchen calendar to remind family members to use less fat, sugar, and salt. − A set of three measuring spoons to measure the amount of fat, sugar, and salt used while cooking.
**2. Information technology**				
A. Internet	• To increase awareness about dietary recommendations and benefits of adapting healthy dietary behaviours • To engage participants actively in the intervention	SMART Eating website (http://smarteating.in/) • Content − Dietary recommendations − Food measurement guide − Health benefits of ‘SMART Eating’ − Tips to increase the consumption of fruits and vegetables − Tips to reduce the consumption of fat, sugar, and salt − Body Mass Index (BMI) − BMI calculator − Quiz − Frequently asked questions (FAQs) Query box	6 months	Website* was available in three languages (English, Hindi and Punjabi). • Content delivery: The content was delivered in parts over three months, with fortnightly addition of new content. • Use of the web site content: Content was used by the participants on their own. • Interactive help: Participants could drop their questions in the query box to discuss any difficulties in using the intervention. The responses to queries were given by the experts in the field of nutrition.
B. Tele-communication		Text messages and email	6 months	Text messages and emails were sent weekly in the English language.
		Social networking app (WhatsApp)		Messages (in the form of text, images and videos) were sent weekly in English, Hindi, and Punjabi.

The comparison group received a pictorial pamphlet on the dietary recommendations of National Institute of Nutrition, India, with information written in Hindi language. Participants were asked to read the pamphlet in their own time, make changes to their diet accordingly, and convey the same information to their family members. The content and pictures in the pamphlet were same as on the dining table mat for intervention group families. One side of the pamphlet had pictorial information on seasonal fruits and vegetables along with dietary recommendations. The other side had pictures of measuring spoons showing the amount of fat, sugar and salt in one spoon as per dietary recommendations; foods high in fat, sugar and salt; and information on how to reduce their intake.

## Data collection

Outcome measurements were made at baseline and six months post-intervention. Baseline data collection took place from June 2015 to March 2016. Data collection tools included: 1) A structured questionnaire to collect socio-demographic data, medical history and physical measurements; 2) A validated food frequency questionnaire (FFQ) “PURE STUDY: FOOD FREQUENCY QUESTIONNAIRE—CHANDIGARH (URBAN), August 2003 ed.”, for individual dietary assessment which had 113 food items [27]; 3) Process evaluation questionnaire including questions for assessing satisfaction with the intervention, household food purchase and consumption [27], and attitude, social influence and self-efficacy.

To account for seasonal variations in the availability of fruits and vegetables, baseline and endline dietary data were collected during the same months. Height was measured to the nearest 0.1 cm using the anthropometric rod. Weight was measured with minimum clothing without shoes to the nearest 0.1 kg on a portable electronic weighing scale (RONMAN Inner Scan Body Composition Monitor, BC-554, TANITA Corporation of America Inc., USA). BMI was calculated as weight (Kg) divided by height (m^2^) [28]. Blood pressure was measured in the sitting position twice at an interval of 10 minutes to the nearest 1 mm Hg, using the electronic OMRON instrument (HM-711DLXCAN, Omran Healthcare, China) [29]

Using standard operating procedures (SOPs), blood samples (5 ml: 1 ml each in EDTA and Oxalate vials, and 3 ml in plain vial) were collected to estimate haemoglobin (g/dl), fasting plasma glucose (mg/dl), total cholesterol (mg/dl), high-density lipoprotein cholesterol (mg/dl), low-density lipoprotein cholesterol (mg/dl) and triglycerides (mg/dl). Instructions regarding overnight (12 hrs.) fasting were given to all the participants one day prior to blood sample collection [30].

## Data analysis

The data analysis was performed using IBM Statistical Package for Social Sciences (SPSS) version 21 and STATA (version 14; College Station, Texas, USA). Intention-to-treat analysis was undertaken for outcome evaluation using ‘Last Observation Carried Forward (LOCF)’ method [31]. Difference-in-differences method (DiD) was used to determine the net change in the outcomes of the intervention group, relative to the comparison group. The underlying assumption of the DiD model is that in the absence of intervention, the difference between intervention and comparison groups is constant over time [32]. Multi-level mixed-effects linear regression models were used to estimate DiD taking into account clustering by introducing cluster-specific random effect in the model. Group (intervention, comparison), time (baseline, endline), and time*group interaction term were included in the model, with the time*group interaction term indicating the intervention effect (differential change) by group from baseline to endline. The net mean change was calculated as the mean change in the intervention group minus the mean change in the comparison group, i.e., DiD. Net relative percentage change in the outcomes was estimated in both the groups as endline mean minus baseline mean divided by the baseline mean and multiplied by 100 [33].

We conducted confirmatory hypothesis tests for the four primary outcomes, i.e., fat, sugar, salt, and fruit and vegetable intake. Therefore, Holm’s adjustment for multiple comparisons was conducted in order to retain a 0.05 family wise type I error rate [34]. The adjusted p-values were 0.0018, 0.0019, 0.0019 and 0.0020, respectively. Multiple hypothesis tests conducted for the secondary outcomes were considered as exploratory analysis; thus, the significance level was retained as 0.05.

The FFQ data were entered into the Spreadsheet software used for the Prospective Urban Rural Epidemiological (PURE) study, India [27] which estimated an individual’s consumption of each food item and the total daily nutrient intakes. Sugar intake was calculated as the sum of sugar present in sweets and packed food (e.g., pasta, rusk, sauce, biscuits, chips) listed in FFQ and the per capita sugar consumption calculated from monthly sugar consumption of the whole family (used at home in tea, milk, coffee, added sugar). Information on sugar content of sweets was obtained from sweet shops, and sugar content of packed food was calculated based on nutrition information labels. Per capita sugar consumption from monthly consumption was calculated by subtracting the amount of total sugar purchased at the beginning of the month and the amount remaining at the end of month and dividing it by 30 days and number of family members [35,36]. Salt intake was calculated by dividing Sodium in mg to 400 (400 mg of Sodium = 1 g salt) [37]. One cup (200 ml) of raw vegetables accounted for 100 g of raw vegetables and one cup (200 ml) of cooked vegetables accounted for 200 g of cooked vegetables. One medium-sized fruit accounted for 100 g of fruit [25].

Digital colorimeter (Labtech Medico Pvt. Ltd., Kerala, India) was used to estimate haemoglobin using Drabkin’s reagent (Cyanmethemoglobin method). Semi-autoanalyser (Erba CHEM 7, Transasia Bio-Medicals Ltd., Mumbai) was used to analyze blood samples using kits provided by the manufacturer. Fasting plasma glucose was estimated by GOD-POD method, Endpoint, total serum cholesterol (CHOD-PAP method, Endpoint), serum triglycerides (GPO-Trinder method, Endpoint), HDL—Cholesterol (Phosphotungstic Acid method, Endpoint) and LDL—Cholesterol (Direct method/Cholesterol—HDL—VLDL).

The process evaluation was initiated at the time of intervention implementation to assess whether the intervention was implemented as per the protocol, to analyse the factors facilitating and hindering the use of the information technology-enabled intervention programme, and to identify points to improve intervention uptake by the participants. Website use was assessed by the number of logins by participants and the visitor count. Use of specific components of the intervention for assessing satisfaction with the program was analysed by summarising the answers to open-ended questions from the process evaluation questionnaire. Other process indicators were: changes in attitude, social influence, and self-efficacy (ASE) score, and monthly purchase and consumption of fat, sugar, salt, and fruit and vegetables.

### Deviation from the protocol

The ‘SMART Eating’ website was password protected but we had to remove the password based on the results of process evaluation as participants found it difficult to login using password and visitor count was added.

## Results

### Participant flow

Of the 732 participants recruited at baseline, the dietary assessment was completed for all. However, 24 participants dropped out before baseline biochemical data collection due to various reasons (unwillingness to provide blood samples, shift in residence, deceased). Therefore, blood sample collection was done among 708 study participants. Forty participants were lost in the six-month follow-up. Thus, 668 (91.3%) participants completed the study at the end-line, indicating a dropout rate of 8.7% (Fig 1), with no significant difference in the percentage of dropout between comparison and intervention groups (9.8% v 7.7%, p = 0.3).

### Baseline characteristics

The baseline characteristics of the study population have been presented in Table 2. The mean age of the participants (n = 732) was 52.7 years, a majority were women (76%), nearly 90% were married, 76% were Hindus, and more than half had completed a college/university degree. The average family monthly income was INR 53968. All socio-economic strata, i.e., low-income, middle- income, and high-income group had equal representation. The average family size was 4.7. Majority of the study participants were non-smokers (93%), and 88% did not consume alcoholic beverages. Sixty percent of the study population was vegetarian. More than 85% of participants were overweight or obese, and about 44% had one or the other medical condition with the prevalence of diabetes and hypertension at 18% and 34%, respectively. No statistically significant differences were observed between the comparison and the intervention groups in terms of socio-demographic characteristics.

**Table 2:** Characteristics of the study participants.

Characteristics		Comparison group (n = 366)	Intervention group (n = 366)	Total (N = 732)
		n (%)	n (%)	n (%)
Age groups, years				
	35–44	88 (24.0)	90 (24.6)	178 (24.3)
	45–54	113 (30.9)	109 (29.8)	222 (30.3)
	55–64	99 (27.7)	108 (29.5)	207 (28.3)
	65 and above	66 (18.0)	59 (16.1)	125 (17.1)
Gender				
	Women	269 (73.5)	288 (78.7)	557 (76.1)
	Men	97 (26.5)	78 (21.3)	175 (23.9)
Marital status				
	Married	329 (89.9)	325 (88.8)	654 (89.3)
	Unmarried	3 (0.8)	2 (0.5)	5 (0.7)
	Widowed/ Separated	34 (9.3)	39 (10.7)	73 (10.0)
Religion				
	Hindu	278 (76.0)	281 (76.8)	559 (76.4)
	Sikh	80 (21.9)	74 (20.2)	154 (21.0)
	Islam	2 (0.5)	0 (0)	2 (0.3)
	Christian	2 (0.5)	3 (0.8)	5 (0.7)
	Others	4 (1.1)	8 (2.2)	12 (1.6)
Education				
	Illiterate	35 (9.6)	44 (12.0)	79 (10.8)
	Primary	18 (4.9)	22 (6.0)	40 (5.5)
	Secondary	25 (6.8)	28 (7.7)	53 (7.2)
	High school	60 (16.4)	58 (15.8)	118 (16.1)
	Higher secondary	35 (9.6)	32 (8.7)	67 (9.2)
	College/University	193 (52.7)	182 (49.7)	375 (51.2)
Occupation				
	Homemakers	226 (61.7)	239 (65.3)	465 (63.5)
	Service	63 (17.2)	50 (13.7)	113 (15.4)
	Business	30 (8.2)	35 (9.6)	65 (8.9)
	Retired	47 (12.8)	42 (11.5)	89 (12.2)
Type of housing				
	Low-Income Group (LIG)	122 (33.3)	122 (33.3)	244 (33.3)
	Middle-Income Group (MIG)	122 (33.3)	122 (33.3)	244 (33.3)
	High-Income Group (HIG)	122 (33.3)	122 (33.3)	244 (33.3)
Family size				
	≤ 4	202 (55.2)	184 (50.3)	386 (52.7)
	> 4	164 (44.8)	182 (49.7)	346 (47.3)
Smoking status				
	Current smokers	14 (3.8)	15 (4.1)	29 (4.0)
	Former smokers	12 (3.3)	14 (3.8)	26 (3.5)
	Never smoked	340 (92.9)	337 (92.1)	677 (92.5)
Alcohol consumption				
	Yes	54 (14.8)	37 (10.1)	91 (12.4)
	No	312 (85.2)	329 (89.9)	641 (87.6)
Food preferences				
	Vegetarian	220 (60.1)	219 (59.8)	439 (60.0)
	Non-vegetarian	146 (39.9)	147 (40.2)	293 (40.0)
Nutrition status (BMI, kg/m^2^)				
	Underweight (<18.5)	6 (1.6)	4 (1.1)	10 (1.4)
	Normal (18.5–22.9)	46 (12.6)	52 (14.2)	98 (13.4)
	Overweight (23.0–24.9)	64 (17.5)	67 (18.3)	131 (17.9)
	Obese (≥25.0)	250 (68.3)	243 (66.4)	493 (67.3)

### Baseline data

Overall, baseline mean fat intake was 86 g/day, salt intake 8.5 g/day, sugar 49 g/day and fruit and vegetables was 368 g/day. At baseline, both macro- and micro-nutrient intakes were similar in both the groups except for vitamin C (234 mg/day v 259 mg/day, p = 0.02). Anthropometric and lipid profile were also comparable in both the groups.

### Outcome evaluation

#### Changes in the dietary intakes

**Fig 3:**
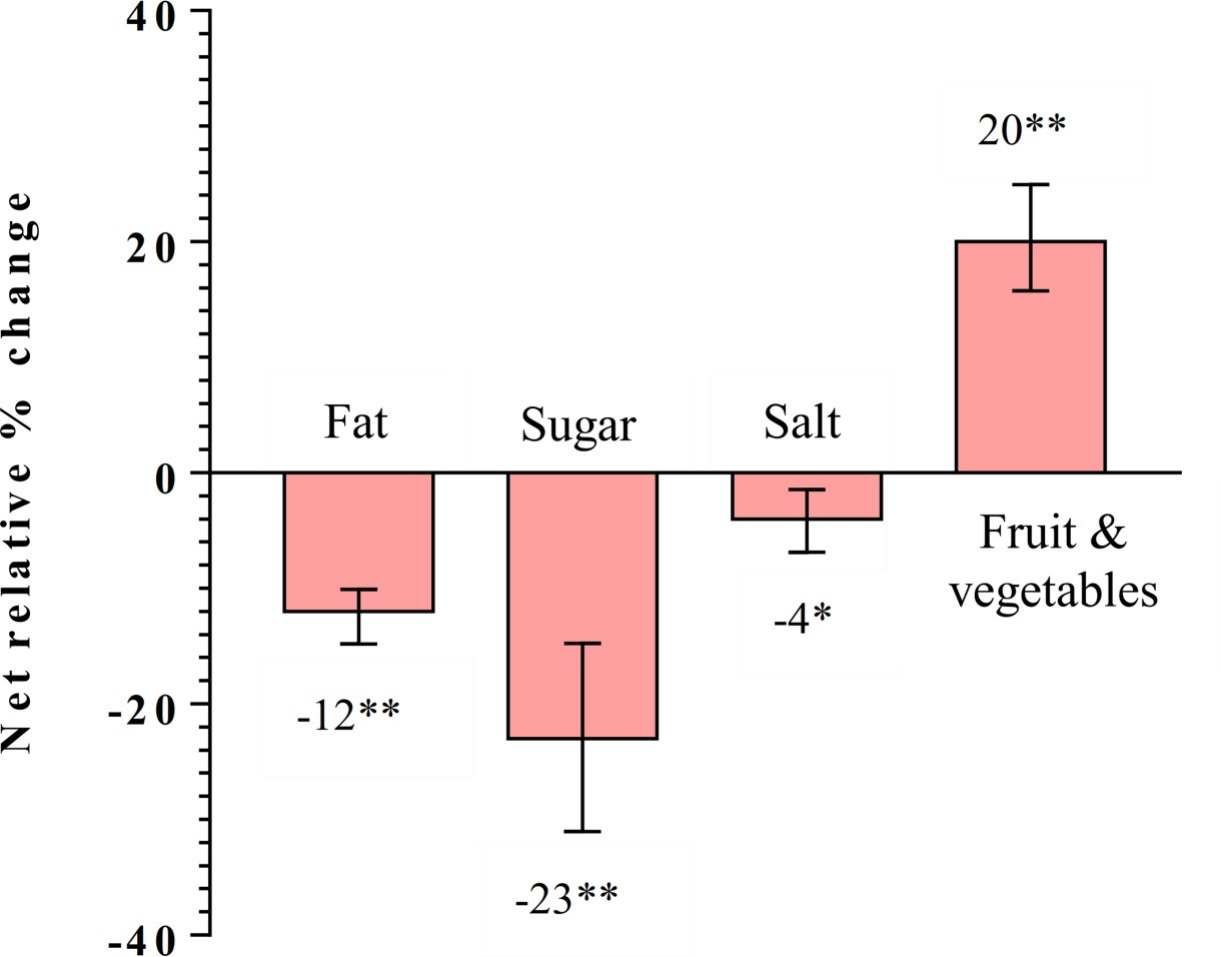
Net relative percentage changes in dietary intakes, *p<0.05, **p<0.001.

The changes from baseline to endline in the mean daily intakes of fat, sugar, salt, fruit and vegetables are presented in Table 3. At the end of 6th month of the intervention, mean fat, sugar, and salt intake reduced significantly in both groups but the magnitude of change was significantly higher in the intervention group compared to the comparison group. However, fruit and vegetable intake reduced significantly in the comparison group but there was a significant increase in the intervention group. The net mean effect, comparing the intervention with the comparison group was, -12.5 g/day for fat (p<0.001), -11.4 g/day for sugar (p<0.001), -0.51 g/day for salt (p<0.001) intake, and +71.6 g/day for fruit and vegetable intake (p<0.001). The net mean effect of the intervention represents a reduction of 12% in fat (95% CI -14.78, -10.06; p<0.001), 23% in sugar (95% CI -31.03, -14.73; p<0.001) and 4% in salt intake (95% CI -7.05, -1.59; p = 0.002), and an increase of 20% in fruit and vegetable intake (95% CI 15.73, 24.93; p<0.001) (Fig 3).

**Table 3:** Changes in dietary intakes of fat, sugar, salt, fruit and vegetables.

Dietary intake (g/day)	Comparison group (n = 366)				Intervention group (n = 366)				Net change^a^	
	Baseline Mean (SE)	Endline Mean (SE)	Mean change (95% CI)	p	Baseline Mean (SE)	Endline Mean (SE)	Mean change^a^ (95% CI)	p	Mean^#^ (95% CI)	p
Fat	86.74 (1.43)	84.99 (1.41)	-1.75 (-3.1, -0.4)	0.01	86.02 (1.57)	71.75 (1.15)	-14.27 (-16.4, -12.2)	<0.001	-12.52 (-15.0, -10.1)	<0.001
Sugar	47.64 (1.33)	45.63 (1.24)	-2.01 (-3.1, -0.9)	<0.001	50.06 (1.40)	36.63 (0.97)	-13.43 (-15.4, -11.5)	<0.001	-11.42 (-13.6, -9.2)	<0.001
Salt	8.49 (0.14)	7.99 (0.13)	-0.50 (-0.7, -0.3)	<0.001	8.44 (0.17)	7.43 (0.13)	-1.01 (-1.2,-0.8)	<0.001	-0.51 (-0.8, -0.2)	<0.001
Fruit & vegetables	357.32 (7.86)	324.71 (6.68)	-32.61 (-43.3, -21.9)	<0.001	379.00 (8.87)	417.96 (8.83)	+38.96 (27.4, 50.5)	<0.001	+71.57 (55.8, 87.3)	<0.001

SE: Standard Error; CI: Confidence Interval

^**a**^Net change = Change in intervention group—Change in comparison group

^**#**^Adjusted for cluster design effect

A significant net effect of the intervention was seen among all the three socioeconomic groups. Significant net reductions were observed in: Fat intake– 14% (p<0.001) in LIG, 13.5% (p<0.001) in MIG and 9% (p<0.001) in HIG; Sugar intake– 22% (p<0.001) in LIG, 29% (p = 0.01) in MIG and 17% (p<0.001) in HIG; and Salt intake– 7.5% (p = 0.002) in LIG, 6% (p = 0.01) in MIG, and 1% (p = 0.7) in HIG. Similarly, a significant net increase was observed in fruit and vegetable intake among all the three socioeconomic groups: 14% (p = 0.001) in LIG, 25.5% (p<0.001) in MIG and 21% (p<0.001) in HIG.

Energy intake reduced significantly in the intervention group, while no change was observed in the comparison group (Table 4). Fibre and vitamin C intake increased in the intervention group whereas it reduced in the comparison group. The net mean effect of the intervention represents a reduction of 10% in energy intake (p<0.001), and 10% in carbohydrate intake. A significant increase of 10% in fibre intake (p<0.001), and increase of 11% in vitamin C intake (p<0.001) was observed in the intervention group compared to the comparison group.

**Table 4:** Changes in energy, macro- and micro-nutrient intakes.

Dietary intake/day	Comparison group (n = 366)				Intervention group (n = 366)				Net change^a^			
	Baseline Mean (SE)	Endline Mean (SE)	Mean change (95% CI)	p	Baseline Mean (SE)	Endline Mean (SE)	Mean change^a^ (95% CI)	p	Mean^#^ (95% CI)	p	% Relative (95% CI)	p
Energy, Kcal	2875 (37.62)	2874 (35.96)	-0.96 (-29.6, 27.7)	0.95	2857 (39.78)	2524 (28.92)	-332.48 (-383.3, -281.6)	<0.001	-331.52 (-389.6, -273.4)	<0.001	-10 (-12.1, -8.6)	<0.001
Carbohydrates, g	439.09 (5.76)	442.64 (5.49)	3.55 (-1.3, 8.4)	0.15	438.22 (6.06)	394.83 (4.56)	-43.39 (-51.4, -35.4)	<0.001	-46.94 (-56.3, -37.6)	<0.001	-10 (-11.5, -7.6)	<0.001
Protein, g	84.42 (1.11)	84.57 (1.07)	0.15 (-0.7, 1.0)	0.74	82.40 (1.16)	74.79 (0.88)	-7.62 (-9.0, -6.2)	<0.001	-7.77 (-9.4, -6.1)	<0.001	-8 (-10.1, -6.5)	<0.001
Fibre, g	13.45 (0.25)	12.75 (0.23)	-0.70 (-1.0, -0.3)	<0.001	14.09 (0.29)	14.50 (0.28)	0.41 (-0.04, 0.9)	0.07	1.11 (0.5, 1.7)	<0.001	10 (6.1, 14.6)	<0.001
Vitamins												
ß-Carotene, μg	2226 (57.58)	2159 (53.17)	-66.67 (-150.2, 16.8)	0.12	2113 (48.62)	2087 (49.25)	-26.46 (-104.9, 52.0)	0.51	40.21 (-73.8, 154.3)	0.49	1 (-4.03, 6.5)	0.65
Vitamin B-12, μg	2.44 (0.06)	2.35 (0.05)	-0.09 (-0.2, -0.02)	0.01	2.37 (0.06)	2.13 (0.05)	-0.24 (-0.3, -0.2)	<0.001	-0.15 (-0.2, -0.05)	<0.01	-7 (-11.5, -2.1)	<0.01
Vitamin C, mg	234.01 (6.81)	219.20 (6.01)	-14.80 (-24.3, -5.4)	<0.01	259.40 (8.42)	264.84 (7.06)	5.44 (-6.2, 17.0)	0.36	20.25 (5.4, 35.1)	0.01	11 (4.4, 16.9)	0.001
Folate, μg	415.50 (5.62)	408.68 (5.24)	-6.82 (-11.8, -1.8)	0.01	411.18 (6.32)	381.74 (4.92)	-29.44 (-36.7, -22.1)	<0.001	-22.62 (-31.4, -13.8)	<0.001	-4 (-6.3, -2.5)	<0.001
Minerals												
Iron, mg	28.14 (0.37)	28.26 (0.36)	0.12 (-0.2, 0.5)	0.47	27.51 (0.40)	25.99 (0.32)	-1.51 (-2.0, -1.0))	<0.001	-1.64 (-2.2, -1.1)	<0.001	-5 (-6.8, -2.5)	<0.001
Sodium, mg	3398 (55.31)	3195 (51.66)	-203.06 (-266.8, -139.3)	<0.001	3377 (66.03)	2973 (50.03)	-404.02 (-490.8, -317.2)	<0.001	-200.96 (-308.2, -93.7)	<0.001	-4 (-6.9, -1.4)	<0.01
Potassium, mg	4111 (64.0)	3993 (57.13)	-118.02 (-181.8, -54.3)	<0.001	4044 (69.97)	3768 (55.11)	-275.86 (-364.5, -187.2)	<0.001	-157.84 (-266.5, -49.2)	<0.01	-3 (-5.2, -0.5)	0.02
Iodine, μg	137.58 (3.10)	131.92 (2.51)	-5.67 (-9.7, -1.6)	0.01	141.81 (3.66)	123.39 (2.41)	-18.42 (-23.4, -13.4)	<0.001	-12.75 (-19.1, -6.3)	<0.001	-8 (-11.6, -4.0)	<0.001

SE: Standard Error; CI: Confidence Interval

^**a**^Net change = Change in intervention group—Change in comparison group

^**#**^Adjusted for cluster design effect

#### Changes in biomarkers

**Fig 4:**
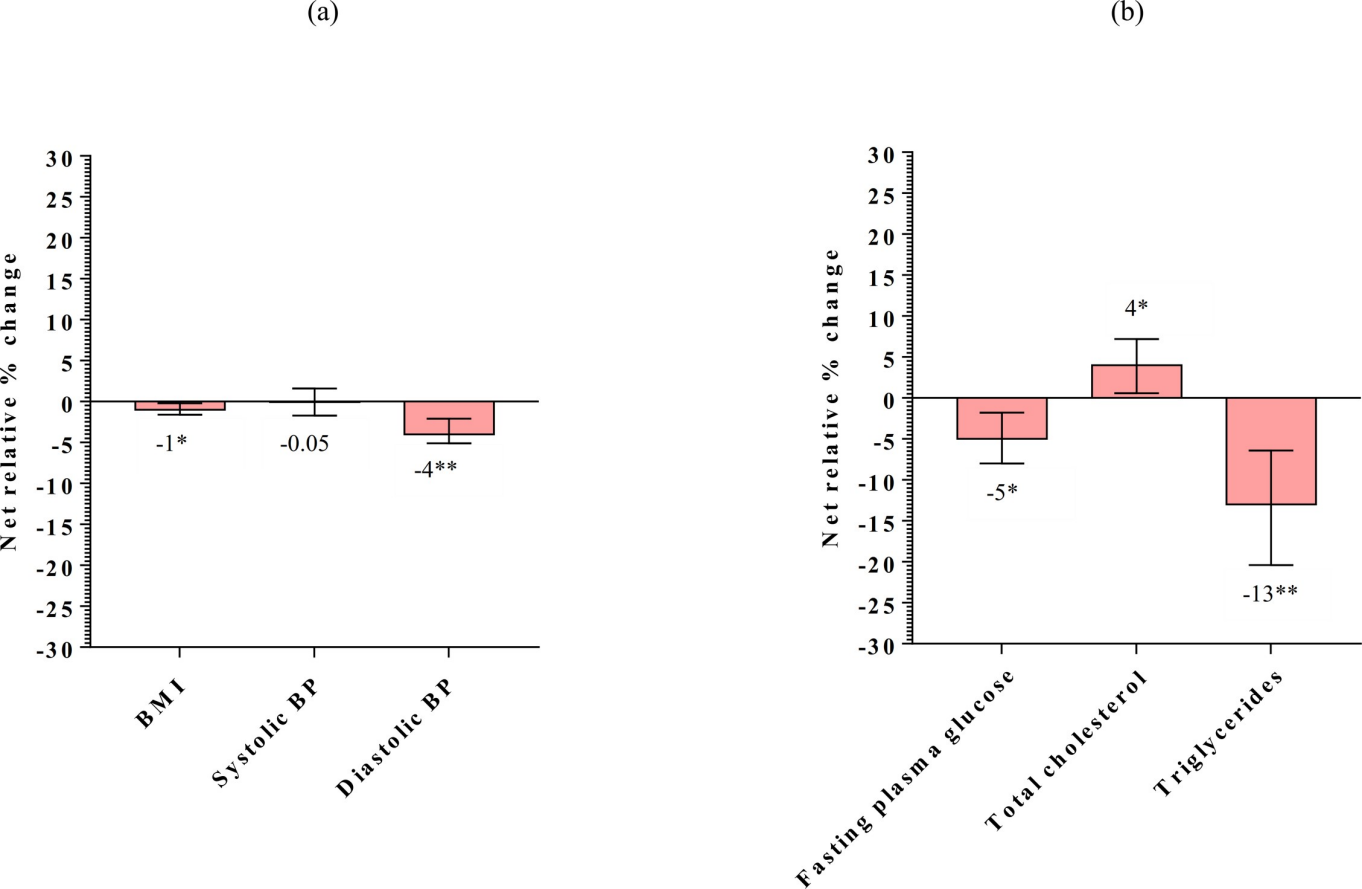
Net relative percentage change in biomarkers: (a) BMI and blood pressure, (b) Fasting plasma glucose and lipids, *p<0.05; **p<0.001; BMI: Body Mass Index; BP: Blood pressure.

The mean net effect of the intervention, when compared with the comparison group, was -0.25 kg/m^2^ (95% CI -0.4, -0.1; p<0.01) on BMI, -2.77 mm Hg (95% CI -4.0, -1.5; p<0.001) on diastolic blood pressure, -5.71 mg/dl (95% CI -10.3, -1.1; p<0.05) on fasting plasma glucose, and -24.20 mg/dl (95% CI -36.0, -12.4; p<0.001) on triglycerides (Table 5). No effect was observed on haemoglobin, high density lipoprotein cholesterol and low density lipoprotein cholesterol while total cholesterol increased (p = 0.04). The mean effect of intervention represents a reduction of 1% in BMI (95% CI -1.6, -0.2; p<0.01), 4% in diastolic blood pressure (95% CI -5.1, -2.1; p<0.001), 5% in plasma glucose levels (95% CI -8.0, -1.8; p<0.01), 13% in triglycerides (95% CI -20.4, -6.4; p<0.001), and 20% in TG/HDL ratio (95% CI -31.1, -8.7; p<0.001), with an increase of 4% in total cholesterol (95% CI 0.6, 7.2; p = 0.02) (Fig 4).

**Table 5:** Changes in biomarkers*.

Clinical characteristics		Comparison group (n = 366)			Intervention group (n = 366)				Net change^a^	
	Baseline Mean (SE)	Endline Mean (SE)	Mean change (95% CI)	p	Baseline Mean (SE)	Endline Mean (SE)	Mean change (95% CI)	P	Mean^#^ (95% CI)	p
Weight, Kg	71.04 (0.71)	71.28 (0.71)	0.24 (-0.1, 0.6)	0.14	69.35 (0.61)	68.92 (0.61)	-0.42 (-0.8, -0.1)	0.01	-0.66 (-1.1, -0.2)	<0.01
BMI, Kg/m^2^	27.45 (0.25)	27.54 (0.25)	0.09 (-0.03, 0.2)	0.15	27.03 (0.22)	26.86 (0.22)	-0.16 (-0.3, -0.03)	0.02	-0.25 (-0.4, -0.1)	<0.01
SBP, mm Hg	135.59 (1.18)	136.85 (1.12)	1.26 (-0.4, 2.9)	0.14	133.15 (1.04)	134.48 (1.02)	1.33 (-0.2, 2.9)	0.10	0.07 (-2.2, 2.3)	0.95
DBP, mm Hg	83.85 (0.69)	85.86 (0.61)	2.01 (1.0, 3.0)	<0.001	83.92 (0.56)	83.15 (0.50)	-0.77 (-1.6, 0.1)	0.07	-2.77 (-4.0, -1.5)	<0.001
Haemoglobin, g/dl	12.66 (0.08)	12.03 (0.08)	-0.64 (0.7, -0.5)	<0.001	12.75 (0.09)	12.10 (0.08)	-0.65 (-0.8, -0.5)	<0.001	-0.01 (-0.2, 0.2)	0.89
FPG, mg/dl	107.07 (2.18)	107.86 (1.99)	0.79 (-2.7, 4.3)	0.66	109.06 (2.41)	104.14 (2.0)	-4.91 (-7.9, -1.9)	<0.001	-5.71 (-10.3, -1.1)	0.01
TC, mg/dl	183.68 (2.13)	180.26 (2.11)	-3.42 (-7.2, 0.4)	0.08	182.88 (2.15)	185.49 (2.22)	2.61 (-1.6, 6.9)	0.23	6.03 (0.3, 11.7)	0.04
HDL-C, mg/dl	43.42 (0.71)	43.69 (0.76)	0.27 (-1.0, 1.6)	0.69	42.65 (0.61)	44.25 (0.62)	1.60 (0.5, 2.7)	<0.01	1.33 (-0.4, 3.1)	0.13
LDL-C, mg/dl	105.15 (1.62)	102.23 (1.51)	-2.92 (-6.0, 0.2)	0.07	107.64 (1.91)	108.96 (1.47)	1.31 (-2.4, 5.0)	0.48	4.23 (-0.6, 9.0)	0.08
Triglycerides, mg/dl	145.16 (4.58)	131.54 (4.15)	-13.63 (-21.7, -5.6)	<0.001	157.54 (6.02)	119.72 (3.97)	-37.82 (-46.5, -29.2)	<0.001	-24.20 (-36.0, -12.4)	<0.001
TC/HDL	4.56 (0.08)	4.52 (0.09)	-0.05 (-0.2, 0.1)	0.54	4.59 (0.08)	4.49 (0.08)	-0.1 (-0.3, 0.1)	0.22	-0.05 (-0.3, 0.2)	0.65
TG/HDL	3.81 (0.16)	3.52 (0.15)	-0.29 (-0.6, -0.02)	0.03	4.27 (0.24)	3.06 (0.13)	-1.21 (-1.6, -0.8)	<0.001	-0.92 (-1.4, -0.5)	<0.001

*N = 708 for blood parameters; SE: Standard Error; CI: Confidence Interval

ªNet change = Change in the intervention group—Change in the comparison group

^**#**^Adjusted for cluster design effect

BMI: Body Mass Index; SBP: Systolic Blood Pressure; DBP: Diastolic Blood Pressure; FPG: Fasting plasma glucose; TC: Total Cholesterol; HDL-C: High density lipoprotein cholesterol; LDL-C: Low density lipoprotein cholesterol; TG: Triglycerides

### Process evaluation

There was a significant increase in ASE score in the intervention group compared to the comparison group. Similarly, a significant reduction in the monthly purchase of fat, sugar, and salt and a significant increase in the monthly purchase of fruits and vegetables, was observed in the intervention group. More than 80% of the participants found the information delivered through IT-enabled intervention (SMS, social networking app, and ‘SMART Eating’ kit) useful, whereas only about 50% of the participants found the website useful. In the comparison group, 43.6% study participants reported that they have read the pamphlets. No specific harms or unintended adverse effects were reported by the participants as a consequence of the trial.

## Discussion

This study examined the effect of information technology-enabled health promotion intervention on dietary behavior compared to traditional education using pamphlets. To the best of our knowledge, this could be the only study aimed at improving dietary behaviour among urban Indian population of diverse socio-economic background using multi-channel communication approach, with the main emphasis on the use of IT. The intervention demonstrated a significant net effect on all four primary outcomes, i.e., fat, sugar, salt, and fruit and vegetable intake.

The context-specific intervention strategies identified through community consultations, guided by the Social Ecological Model and PRECEDE-PROCEED planning model, active engagement of the participants through social networking app with prompt response to their queries, and the use of multi-channel communication approaches, could have resulted in significant net effect on primary as well as some of the secondary outcomes. In addition to emphasis on consuming seasonal fruits and vegetables, involvement of family members in intervention implementation, and provision of ‘SMART Eating’ kit could have improved self-efficacy of the families in bringing dietary changes.

There is a lack of similar interventions in the Indian context. A community-based study from rural South India, using traditional methods of education for increasing fruit and vegetable intake and reducing monthly household consumption of oil, sugar, and salt, demonstrated no net effect on fruit and vegetable intake. The authors attributed no effect to intervention contamination due to exposure to information to the comparison group through other NCD prevention programmes [38]. In the present study, in addition to other intervention strategies, the availablity of fruits and vegetables in the study area close to their living place could have been a strong facilitator for increasing the intake, which was reported as a major barrier in rural setting [38]. A mobile phone text message intervention for improving diabetes risk behaviors, reported improvement in health behavior composite score (fat, and fruit and vegetable intake), but the outcome measures were based on subjective self-reprted consumption rather than objective measurement [17]. Therefore, the magnitude of the effect of the intervention was not very clear.

Intervention studies on fat, sugar, salt, fruit and vegetable intake among adults have been conducted across the globe among diverse populations and settings. These include high risk groups [39,40] primary care settings [41,42], workplace [43]; and community settings [44,45]. The efficacy of these dietary interventions showed varied results for the primary and the secondary outcomes. These differences could be due to differences in the number of dietary components chosen, methods of intervention implementation, duration of the intervention, varied methods used for outcome evaluation, and contamination of interventions due to exposure to other public health interventions during the study period.

In comparison to other studies, the present study had higher effect on fat and sugar intake [45]. This could be because the intervention emphasised that reducing the intake of fat and sugar could save money, which in turn can be used to buy fruits and vegetables. The net effect of the ‘SMART Eating’ intervention on dietary salt intake was lower compared to an exclusive salt reduction intervention reported by Takahashi *et al*. (2003) in Japan, which used two individual counseling sessions by a dietitian, two newsletters, a group lecture and computer tailored education [46]. The possible explanation could be low self-efficacy in changing the taste developed over the years for food high in salt which was a major concern raised by participants during formative research. The intervention had a similar effect on increasing fruit and vegetable intake as reported from developed countries using traditional methods of education [44,47] and mobile technology [48]. In contrast, a lifestyle intervention in Sweden using counseling, group discussions on healthy eating by health workers and cooking classes by a chef to increase fruit and vegetable intake and to reduce fat intake, could not produce a significant change in behaviour outcomes [49]. Similarly, a 9-months web-based computer-tailored intervention aimed at increasing fruit and vegetable intake and decreasing saturated fat intake, did not result in significant intervention effects among Netherland adults [50]. Compared to the present study, interventions on reducing fat and sugar intake demonstrated higher effect on weight reduction [13,51]. Consistent with our findings, most of the interventions could not produce reduction in SBP [44,51]; however, a few interventions reported an increase in both SBP and DBP [52]. Diastolic blood pressure reduced significantly in the present study which is an indicator of salt sensitivity [53] and has been shown to be related to urinary sodium excretion in hypertensive populations [54]. In contrast to our ‘SMART Eating’ intervention, low-fat diet interventions did not improve any of the lipid parameters and plasma glucose [44,51]. High carbohydrate intake lowers TC and LDL-C [55,56]. Hence, reduction in carbohydrate intake in the present study could have resulted in an increase in TC.

### Methodological considerations

We used food frequency questionnaire (FFQ) for dietary assessments which had been developed and validated in a northern Indian setting [27]. However, FFQs are prone to overestimation compared to other methods [57]. Sources of errors (random and systematic) are inherent in dietary assessments methods which may lead to biases towards or away from the null hypothesis. Self-reported dietary intake may also have recall bias which could underestimate actual intake [58]. There could also be the possibility of social desirability bias leading to overestimation of the effect size. However, net change observed in objective measurements (anthropometrics, blood pressure, and serum lipids) indicates that these biases did not affect this study.

In the present study, the sample size calculation was estimated based on qualitative outcome variables, i.e., the percentage of participants meeting dietary recommendations. As most of the outcome variables were quantitative, changes in the outcomes were estimated quantitatively as this approach is more efficient in measuring the intervention effects. The study had adequate power to estimate the effects. Therefore, quantitative outcome estimates of the present study can be used to estimate sample size in future studies. The sample size in the present study was based on an assumption of 20% improvement in the primary dietary outcomes which were observed for sugar (23%), and fruit and vegetable (20%) intake behaviours but improvement of similar magnitude were not observed for fat (12%) and salt intake behaviours (4%), though these changes were statistically significant. We have done Holm’s adjustment for multiple comparisons to alpha level for primary outcomes (Table 3). Beside the hypothesis testing for primary outcomes, several statistical tests have been conducted for secondary outcomes and sub-groups which should be considered as exploratory analysis.

The loss to follow-up is often hard to avoid in randomized trials. In the present study, those who did or did not complete the study did not differ in their baseline characteristics, except gender; higher percentage of men dropped out compared to women (12.6% v 7.5%, p = 0.04). Intention-to-treat (ITT) analysis was performed by the inclusion of all participants in data analysis to reduce selection bias and have an unbiased estimation of the effect [59]. ITT minimizes type I error and gives an unbiased estimate of the treatment effect because of dilution caused by noncompliance [60,61]. Analysis was adjusted for cluster design effect using multi-level mixed effects linear regression models. As there were no significant differences in baseline characteristics between the comparison and the intervention groups, adjustment of confounders was not needed in the context of a cluster RCT. However, difference-in-differences (DiD) method was used to account for unmeasured confounders, if any.

### Strengths and limitations

The main strength of this study is the inclusion of all socio-economic groups which has enhanced the generalisability. The calculated sample size is large compared to other RCTs, which provides adequate power to the study. Strict randomization throughout the sampling procedure has also helped in minimization of any potential confounding bias, although the lack of blinding means the results should be interpreted with caution.

Main limitation of the study was the selection of only one member from each family to measure dietary changes, as it was impractical to measure dietary intake of all members of the family which may underestimate the effect size. Low male participation in the trial was another limitation of this study. It seems that in India, women are responsible for preparation diets so their participation was more. Similar findings have been reported by a review study [62], and other similar studies from India [63,64]. However, the involvement of males (especially husbands and sons) as co-champions in intervention implementation at the family level had increased their participation. The sample size was doubled to do analysis by two subgroups. However, we had three socioeconomic subgroups, so the sample size should have been trebled. Therefore, socioeconomic subgroup analysis should be considered as exploratory analysis.

The multi-channel communication approach for delivering the IT-enabled nutrition intervention is unique. The intervention made use of available IT tools which posed no additional burden on the families. Visitor count was used as an indicator of use of website content by the participants. However, due to lack of resources, it was not possible to measure the time spent on the website. The process evaluation undertaken during intervention implementation indicated that password protection of the website makes it difficult for the users to log-in. This helped in taking remedial measures to improve dose of the intervention received by the participants. Majority of the participants (more than 90%) used ‘SMS’, social networking app and ‘SMART Eating’ kit articles, which was another strength of the present study.

This study provided important information on the feasibility, acceptability, and effectiveness of multi-strategy intervention using information technology in bringing about dietary behaviour change in a developing country but the comparative effectiveness of each component of the intervention was not undertaken due to resource constraints.

### Implications

The knowledge of dietary recommendations, improving awareness of dietary guidelines and associated health risks, all are important for bringing change in diets, therefore, efforts are needed to translate written documents of dietary guidelines into practical applications using IT for mass media. However, knowledge alone may not work, there is a need to facilitate the change process by enhancing people’s self-efficacy for initiating changes through reminders, for which IT could be the best solution. There is a need to focus on fat, sugar, and salt reduction which does not involve cost and thus it is feasible to implement these interventions even among low-income groups. Therefore, to remove inequality, future interventions can strongly consider working with people from all socio-economic strata. However, specific measures for improving fruit and vegetable intake through improved access are required in the light of the increasing prices of fruits and vegetables. Unhealthy dietary behaviours cluster; thus, prevention of chronic diseases requires modifications in multiple dietary behaviours. Therefore, future interventions can focus on engaging populations in adapting multiple healthy behaviours, involving family members especially males, who would provide a supportive environment for enabling maintenance of healthy behaviours. The exploratory analysis conducted for secondary outcomes and sub-groups in this study could be used as a guide for hypothesis building in future studies.

Study findings have major public health implications. In India, although control of chronic diseases is managed within the health care systems, however, healthcare services currently have low capacity to focus efforts on health promotion due to lack of resources. The use of IT as a health promotion strategy for future interventions in different settings may prove successful because it removes the limitations of resources and geographical distances especially for low-income strata populations. Similar trials based on multi-channel communication approach using IT tools should also be planned to test the effectiveness of such interventions among rural populations as IT has high penetration in these areas too. Given that behaviour change is a difficult and complex process; further work is needed to determine the sustainability of intervention effect along with exploratory research on understanding barriers to sustainability. The present study demonstrated the potential for population level behaviour change in a low middle-income country. The findings of this study could be implemented in different settings in India and in other low-income countries with modifications to account for contextual differences.

## Conclusions

The ‘SMART Eating’ intervention delivered using multi-channel communication approaches, was effective in improving dietary behaviours among urban adults from diverse socio-economic backgrounds. Context specific intervention strategies identified through formative research guided by Social Ecological Model and systematic development of the intervention guided by PRECEDE-PROCEED model, enabled significant net reduction in fat, sugar and salt intake, and significant net increase in fruit and vegetable intake in the intervention group. Availability of fruits and vegetables in the study area was one of the facilitating factors for improving the intake. The intervention was also successful in reducing weight, maintaining systolic blood pressure, reducing diastolic blood pressure, fasting plasma glucose, and triglycerides. Overall, the study demonstrated the feasibility and acceptability of IT-enabled nutrition education intervention among urban population. The study indicated that it is possible to integrate efforts on changing multiple behaviours rather than focussing on single behaviour change. However, the effectiveness of this comprehensive intervention package, tested in a controlled setting, needs to be further explored through implementation research before its potential scale up.

## Supporting information

S1 CONSORT Checklist Effectiveness of IT-enabled intervention for ‘SMART Eating’: A cluster randomized trial. (PDF)

S1 Dataset (SAV)

## References

[1] World Health Organization Diet, nutrition and the prevention of chronic diseases: report of a Joint WHO/FAO Expert Consultation WHO Technical Report Series 916. Geneva: World Health Organization; 2003. 12768890

[2] World Health Organization Global Strategy on Diet, Physical Activity and Health. Geneva: World Health Organization; 2004.

[3] NACO. Annual Report—2016–17. National AIDS Control Organization (NACO). New Delhi: 2017.

[4] World Health Organization. Non-communicable diseases—Fact sheet. 2018 [cited 2018 May 30]. Available from: http://www.who.int/en/news-room/fact-sheets/detail/noncommunicable-diseases.

[5] Meenakshi JV. Trends and patterns in the triple burden of malnutrition in India. Agricultural Economics. 2016;47:115–34. 10.1111/agec.12304

[6] International Institute for Population Sciences (IIPS) and ICF. National Family Health Survey (NFHS-4), 2015–16: India. Mumbai: International Institute for Population Sciences; 2017.

[7] National Institute of Nutrition (NIN), India. Diet and Nutrition Status of Urban Population of India: Report on Diet and Nutritional Status of Urban Population and prevalence of obesity, hypertension, diabetes and its associated non-communicable diseases released by NIN. 2017 [cited 2018 Feb 22]. Available from: http://icmr.nic.in/icmrnews/nin/ANNEXURE%20TO%20MEDIA%20RELEASE.pdf.

[8] National White Paper (3rd Edition). Synergizing Efforts in Diabetes Care at the Tertiary Level—Strengthening Policies and Practices around Diabetes Management. A joint initiative of Confederation of India Industry, Ministry of Health and Family Welfare Government of India, the Lilly NCD partnership and Knowledge partner psi India. http://www.psi.org/wp-content/uploads/2015/09/National-NCD-White-Paper-2015.pdf. Accessed 20 Aug 2017.

[9] World Health Organization. Diet and Physical Activity: a public health priority. http://www.who.int/dietphysicalactivity/background/en/. Accessed 25 Aug 2017.

[10] Contento I, Balch GI, Bronner YL, Lytle LA, Maloney SK, Olson CM, et al The effectiveness of nutrition education and implications for nutrition education policy, programs, and research: a review of research. J Nutr Educ. 1995;27(6):227–418.

[11] Pomerleau J, Lock K, Knai C, McKee M. Interventions designed to increase adult fruit and vegetable intake can be effective: a systematic review of the literature. J Nutr. 2005;135(10):2486–95. 10.1093/jn/135.10.2486 16177217

[12] Krebs P, Prochaska JO, Rossi JS. A meta-analysis of computer-tailored interventions for health behavior change. Prev Med. 2010;51(3–4):214–21. 10.1016/j.ypmed.2010.06.004 20558196 PMC2939185

[13] Jaime PC, Bandoni DH, Sarno F. Impact of an education intervention using email for the prevention of weight gain among adult workers. Public Health Nutr. 2014;17(7):1620–7. 10.1017/S1368980013001936 23962422 PMC10282498

[14] Afshin A, Babalola D, McLean M, Yu Z, Ma W, Chen CY, et al Information Technology and Lifestyle: A Systematic Evaluation of Internet and Mobile Interventions for Improving Diet, Physical Activity, Obesity, Tobacco, and Alcohol Use. Journal of the American Heart Association. 2016;5(9):e003058 10.1161/JAHA.115.003058 27581172 PMC5079005

[15] Harris J, Felix L, Miners A, Murray E, Michie S, Ferguson E, et al Adaptive e-learning to improve dietary behaviour: a systematic review and cost-effectiveness analysis. Health Technol Assess. 2011;15(37):1–160. 10.3310/hta15370 22030014 PMC4781244

[16] Feinberg L, Menon J, Smith R, Rajeev JG, Kumar RK, Banerjee A. Potential for mobile health (mHealth) prevention of cardiovascular diseases in Kerala: A population-based survey. Indian Heart J. 2017;69(2):182–99. 10.1016/j.ihj.2016.11.004 28460766 PMC5414958

[17] Pfammatter A, Spring B, Saligram N, Dave R, Gowda A, Blais L, et al mHealth Intervention to Improve Diabetes Risk Behaviors in India: A Prospective, Parallel Group Cohort Study. J Med Internet Res. 2016;18(8):e207 10.2196/jmir.5712 27496271 PMC4992169

[18] Svetkey LP, Batch BC, Lin PH, Intille SS, Corsino L, Tyson CC, et al Cell phone Intervention for You (CITY): A randomized, controlled trial of behavioral weight loss intervention for young adults using mobile technology. Obesity. 2015;23(11):2133–41. 10.1002/oby.21226 26530929 PMC4636032

[19] Campbell MK, Piaggio G, Elbourne DR, Altman DG. Consort 2010 statement: extension to cluster randomised trials. BMJ: British Medical Journal. 2012;345. 10.1136/bmj.e5661 22951546

[20] Kaur J, Kaur M, Webster J, Kumar R. Protocol for a cluster randomised controlled trial on information technology-enabled nutrition intervention among urban adults in Chandigarh (India): SMART eating trial. Global Health Action. 2018;11(1):1419738 10.1080/16549716.2017.1419738 29370744 PMC5795704

[21] Land MA, Webster J, Christoforou A, Praveen D, Jeffery P, Chalmers J, et al Salt intake assessed by 24 h urinary sodium excretion in a random and opportunistic sample in Australia. BMJ open. 2014;4:e003720 10.1136/bmjopen-2013-003720 24440795 PMC3902305

[22] Hayes RJ, Bennett S. Simple sample size calculation for cluster-randomized trials. Int J Epidemiol. 1999;28(2):319–26. 10.1093/ije/28.2.319 10342698

[23] Fewtrell MS, Kennedy K, Singhal A, Martin RM, Ness A, Hadders-Algra M, et al How much loss to follow-up is acceptable in long-term randomised trials and prospective studies? Arch Dis Child. 2008;93(6):458–61. 10.1136/adc.2007.127316 18495909

[24] Clark CC, Paraska KK. Chapter 2, Concepts, Models, and Theories In: Health Promotion for Nurses: A Practical Guide. USA: Johns and Bartlett Learning; 2014.

[25] National Institute of Nutrition. Indian Council of Medical Research Dietary guidelines for Indians: A manual. Hyderabad, India: ICMR; 2011.

[26] Kaur J, Kaur M, Chakrapani V, Kumar R. Multi-level influences on dietary behaviours among urban Indians: Application of the Social Ecological Model. SAGE Open.

[27] Mahajan R, Malik M, Bharathi AV, Lakshmi PV, Patro BK, Rana SK, et al Reproducibility and validity of a quantitative food frequency questionnaire in an urban and rural area of northern India. Natl Med J India. 2013;26(5):266–72. 25017832

[28] Tsai AC, Burns BF. Syndemics of psychosocial problems and HIV risk: A systematic review of empirical tests of the disease interaction concept. Soc Sci Med. 2015;139:26–35. 10.1016/j.socscimed.2015.06.024 26150065 PMC4519429

[29] Pickering TG, Hall JE, Appel LJ, Falkner BE, Graves J, Hill MN, et al AHA Scientific Statement: Recommendations for blood pressure measurement in humans and experimental animals—Part 1: Blood pressure measurement in humans. Circulation. 2005;111(5):697–716. 10.1161/01.CIR.0000154900.76284.F6 15699287

[30] World Health Organisation. WHO STEPS surveillance manual: The WHO STEPwise approach to chronic disease risk factor surveillance. 2005 [cited 2017 Aug 28]. Available from: http://apps.who.int/iris/bitstream/10665/43376/1/9241593830_eng.pdf.

[31] White IR, Carpenter J, Horton NJ. Including all individuals is not enough: lessons for intention-to-treat analysis. Clin Trials. 2012;9(4):396–407. 10.1177/1740774512450098 22752633 PMC3428470

[32] Mustanski B, Garofalo R, Herrick A, Donenberg G. Psychosocial health problems increase risk for HIV among urban young men who have sex with men: preliminary evidence of a syndemic in need of attention. Ann Behav Med. 2007;34(1):37–45. 10.1080/08836610701495268 17688395 PMC2219199

[33] Bhardwaj S, Misra A, Gulati S, Anoop S, Kamal VK, Pandey RM. A randomized controlled trial to evaluate the effects of high Protein Complete (lActo) VEgetaRian (PACER) diet in non-diabetic obese Asian Indians in North India. Heliyon. 2017;3(12):e00472 10.1016/j.heliyon.2017.e00472 29387815 PMC5772352

[34] Chen S-Y, Feng Z, Yi X. A general introduction to adjustment for multiple comparisons. J Thorac Dis. 2017;9(6):1725–9. 10.21037/jtd.2017.05.34 28740688 PMC5506159

[35] UNIT 8 Assessment of nutritional status in community settings -11 [cited 2017 Nov 15]. Available from: http://egyankosh.ac.in/bitstream/123456789/33465/1/Unit-8.pdf.

[36] Shrivastava S, Shrivastava P, Ramasamy J. Assessment of nutritional status in the community and clinical settings. J Med Sci. 2014;34(5):211–3.

[37] Mohan S, D. P. World Health Organization. Technical paper—Review of salt and health: Situation in South-East Asia Region. Technical working group meeting on regional action plan and targets for prevention and control of NCDs Bangkok, Thailand, 11–13 June 2013. [cited 2018 Feb 20]. Available from: http://www.searo.who.int/entity/noncommunicable_diseases/events/ncd_twg_bangkok_technical_paper_review_of_salt_and_health.pdf.

[38] Daivadanam M, Wahlström R, Ravindran TKS, Sarma PS, Sivasankaran S, Thankappan KR. Changing household dietary behaviours through community-based networks: A pragmatic cluster randomized controlled trial in rural Kerala, India. PLoS One. 2018;13(8):e0201877 10.1371/journal.pone.0201877 30133467 PMC6104953

[39] Wright JL, Sherriff JL, Dhaliwal SS, Mamo JC. Tailored, iterative, printed dietary feedback is as effective as group education in improving dietary behaviours: results from a randomised control trial in middle-aged adults with cardiovascular risk factors. The international journal of behavioral nutrition and physical activity. 2011;8:43 10.1186/1479-5868-8-43 21595978 PMC3117757

[40] Djuric Z, Poore KM, Depper JB, Uhley VE, Lababidi S, Covington C, et al Methods to increase fruit and vegetable intake with and without a decrease in fat intake: compliance and effects on body weight in the nutrition and breast health study. Nutr Cancer. 2002;43(2):141–51. 10.1207/S15327914NC432_4 12588694

[41] John JH, Ziebland S, Yudkin P, Roe LS, Neil HA, Oxford F, et al Effects of fruit and vegetable consumption on plasma antioxidant concentrations and blood pressure: a randomised controlled trial. Lancet. 2002;359(9322):1969–74. 10.1016/s0140-6736(02)98858-6 12076551

[42] Lanza E, Schatzkin A, Daston C, Corle D, Freedman L, Ballard-Barbash R, et al Implementation of a 4-y, high-fiber, high-fruit-and-vegetable, low-fat dietary intervention: results of dietary changes in the Polyp Prevention Trial. Am J Clin Nutr. 2001;74(3):387–401. 10.1093/ajcn/74.3.387 11522565

[43] Engbers LH, van Poppel MN, Chin APM, van Mechelen W. The effects of a controlled worksite environmental intervention on determinants of dietary behavior and self-reported fruit, vegetable and fat intake. BMC Public Health. 2006;6:253 10.1186/1471-2458-6-253 17044935 PMC1626462

[44] Kennedy BM, Katzmarzyk PT, Johnson WD, Johnson GS, McGee BB, Champagne CM, et al People United to Sustain Health (PUSH): a community-based participatory research study. Clin Transl Sci. 2014;7(2):108–14. 10.1111/cts.12133 24405579 PMC4006294

[45] Paineau DL, Beaufils F, Boulier A, Cassuto DA, Chwalow J, Combris P, et al Family dietary coaching to improve nutritional intakes and body weight control: a randomized controlled trial. Arch Pediatr Adolesc Med. 2008;162(1):34–43. 10.1001/archpediatrics.2007.2 18180410

[46] Takashashi Y, Sasaki S, Takahashi M, Okubo S, Hayashi M, Tsugane S. A population-based dietary intervention trial in a high-risk area for stomach cancer and stroke: changes in intakes and related biomarkers. Prev Med. 2003;37(5):432–41. 10.1016/s0091-7435(03)00164-6 14572428

[47] Greene GW, Fey-Yensan N, Padula C, Rossi SR, Rossi JS, Clark PG. Change in fruit and vegetable intake over 24 months in older adults: results of the SENIOR project intervention. Gerontologist. 2008;48(3):378–87. 10.1093/geront/48.3.378 18591363

[48] Stevens VJ, Glasgow RE, Toobert DJ, Karanja N, Smith KS. One-year results from a brief, computer-assisted intervention to decrease consumption of fat and increase consumption of fruits and vegetables. Prev Med. 2003;36(5):594–600. 10.1016/s0091-7435(03)00019-7 12689805

[49] Siddiqui F, Winther V, Kurbasic A, Sonestedt E, Lundgren KB, Lindeberg S, et al Changes in dietary intake following a culturally adapted lifestyle intervention among Iraqi immigrants to Sweden at high risk of type 2 diabetes: a randomised trial. Public Health Nutr. 2017;20(15):2827–38. 10.1017/S136898001700146X 28738912 PMC10261524

[50] Springvloet L, Lechner L, de Vries H, Oenema A. Long-term efficacy of a Web-based computer-tailored nutrition education intervention for adults including cognitive and environmental feedback: a randomized controlled trial. BMC Public Health. 2015;15:372 10.1186/s12889-015-1707-4 25887891 PMC4424509

[51] Bazzano LA, Hu T, Reynolds K, Yao L, Bunol C, Liu Y, et al Effects of low-carbohydrate and low-fat diets: a randomized trial. Ann Intern Med. 2014;161(5):309–18. 10.7326/M14-0180 25178568 PMC4428290

[52] He FJ, Wu Y, Feng XX, Ma J, Ma Y, Wang H, et al School based education programme to reduce salt intake in children and their families (School-EduSalt): cluster randomised controlled trial. BMJ. 2015;350:h770 10.1136/bmj.h770 25788018 PMC4364292

[53] Costa Ede A, Rose G, Klein CH, Achutti AC. Diastolic pressure as an index of salt sensitivity. J Hum Hypertens. 1994;8(9):703–9. 7807501

[54] Cheung BMY, Ho SPC, Cheung AHK, Lau CP. Diastolic blood pressure is related to urinary sodium excretion in hypertensive Chinese patients. Q J Med. 2000;93(3):163–8. 10.1093/qjmed/93.3.163 10751235

[55] Dehghan M, Mente A, Zhang X, Swaminathan S, Li W, Mohan V, et al Associations of fats and carbohydrate intake with cardiovascular disease and mortality in 18 countries from five continents (PURE): a prospective cohort study. Lancet. 2017;390(10107):2050–62. 10.1016/S0140-6736(17)32252-3 28864332

[56] Jung CH, Choi KM. Impact of High-Carbohydrate Diet on Metabolic Parameters in Patients with Type 2 Diabetes. Nutrients. 2017;9(4):E322 10.3390/nu9040322 28338608 PMC5409661

[57] Steinemann N, Grize L, Ziesemer K, Kauf P, Probst-Hensch N, Brombach C. Relative validation of a food frequency questionnaire to estimate food intake in an adult population. Food & nutrition research. 2017;61(1):1305193. 28469546 10.1080/16546628.2017.1305193 PMC5404419

[58] Naska A, Lagiou A, Lagiou P. Dietary assessment methods in epidemiological research: current state of the art and future prospects. F1000Research. 2017;6:926 10.12688/f1000research.10703.1 28690835 PMC5482335

[59] Heritier SR, Gebski VJ, Keech AC. Inclusion of patients in clinical trial analysis: the intention-to-treat principle. Med J Aust. 2003;179(8):438–40. 14558871 10.5694/j.1326-5377.2003.tb05627.x

[60] Fergusson D, Aaron SD, Guyatt G, Hebert P. Post-randomisation exclusions: the intention to treat principle and excluding patients from analysis. BMJ. 2002;325(7365):652–4. 10.1136/bmj.325.7365.652 12242181 PMC1124168

[61] Gupta SK. Intention-to-treat concept: A review. Perspect Clin Res. 2011;2(3):109–12. 10.4103/2229-3485.83221 21897887 PMC3159210

[62] Pagoto SL, Schneider KL, Oleski JL, Luciani JM, Bodenlos JS, Whited MC. Male inclusion in randomized controlled trials of lifestyle weight loss interventions. Obesity. 2012;20(6):1234–9. 10.1038/oby.2011.140 21633403

[63] Daivadanam M, Wahlstrom R, Ravindran TKS, Sarma PS, Sivasankaran S, Thankappan KR. Design and methodology of a community-based cluster-randomized controlled trial for dietary behaviour change in rural Kerala. Global Health Action. 2013;6(1):20993. 23866917 10.3402/gha.v6i0.20993 PMC3715653

[64] Thankappan K, Shah B, Mathur P, Sarma P, Srinivas G, Mini G, et al Risk factor profile for chronic non-communicable diseases: results of a community-based study in Kerala, India. Indian J Med Res. 2010;131(1):53–63. 20167974

