# Supplementary material for: Effectiveness of information technology–enabled ‘SMART Eating’ health promotion intervention: A cluster randomized controlled trial
Source: PLoS One. 2020 Jan 10;15(1):e0225892. doi: 10.1371/journal.pone.0225892 (PMC6953835; doi:10.1371/journal.pone.0225892)
Supplement: S1 CONSORT Checklist — (PDF) [file pone.0225892.s001.pdf]

Supporting information

S1 CONSORT Checklist. **Effectiveness of IT-enabled intervention for 'SMART Eating': A cluster randomized trial**

Section/Topic	Item No	Checklist item	Reported on page No
**Title and abstract**			
	1a	Identification as a randomised trial in the title: Cluster randomized trial	1 [Title]
	1b	Structured summary of trial design, methods, results, and conclusions (for specific guidance see CONSORT for abstracts)	2-3 [Abstract]
**Introduction**			
Background and objectives	2a	Scientific background and explanation of rationale	4-5 [Background]
	2b	Specific objectives or hypotheses	5 [Background]
**Methods**			
Trial design	3a	Description of trial design (such as parallel, factorial) including allocation ratio	5, 6 [Study design]
	3b	Important changes to methods after trial commencement (such as eligibility criteria), with reasons	12 [Deviation from protocol]
Participants	4a	Eligibility criteria for participants	6 [Inclusion & exclusion criteria]
	4b	Settings and locations where the data were collected	5-6 [Study setting]
Interventions	5	The interventions for each group with sufficient details to allow replication, including how and when they were actually administered	7-9 [Intervention description]
Outcomes	6a	Completely defined pre-specified primary and secondary outcome measures, including how and when they were assessed	7 [Study outcomes], 10 [Data collection]
	6b	Any changes to trial outcomes after the trial commenced, with reasons	-
Sample size	7a	How sample size was determined	6-7 [Sample size]
	7b	When applicable, explanation of any interim analyses and stopping guidelines	-
Randomisation:			
Sequence generation	8a	Method used to generate the random allocation sequence	6 [Sampling procedure]
	8b	Type of randomisation; details of any restriction (such as blocking and block size)	6 [Sampling procedure]
Allocation concealment mechanism	9	Mechanism used to implement the random allocation sequence (such as sequentially numbered containers), describing any steps taken to conceal the sequence until interventions were assigned	6 [Sampling procedure]
Implementation	10	Who generated the random allocation sequence, who enrolled participants, and who assigned participants to interventions	6 [Sampling procedure]
Blinding	11a	If done, who was blinded after assignment to interventions (for example, participants, care providers, those assessing	-

		outcomes) and how	
	11b	If relevant, description of the similarity of interventions	-
Statistical methods	12a	Statistical methods used to compare groups for primary and secondary outcomes	10-12 [Data analysis]
	12b	Methods for additional analyses, such as subgroup analyses and adjusted analyses	10-12 [Data analysis]
**Results**			
Participant flow (a diagram is strongly recommended)	13a	For each group, the numbers of participants who were randomly assigned, received intended treatment, and were analysed for the primary outcome	12 [Flow diagram]
	13b	For each group, losses and exclusions after randomisation, together with reasons	12 [Flow diagram]
Recruitment	14a	Dates defining the periods of recruitment and follow-up	10 [Data collection]
	14b	Why the trial ended or was stopped	10 [Data collection]
Baseline data	15	A table showing baseline demographic and clinical characteristics for each group	12-14 [Baseline characteristics]
Numbers analysed	16	For each group, number of participants (denominator) included in each analysis and whether the analysis was by original assigned groups	12 [Flow diagram]
Outcomes and estimation	17a	For each primary and secondary outcome, results for each group, and the estimated effect size and its precision (such as 95% confidence interval)	14-19 [Outcome evaluation]
	17b	For binary outcomes, presentation of both absolute and relative effect sizes is recommended	-
Ancillary analyses	18	Results of any other analyses performed, including subgroup analyses and adjusted analyses, distinguishing pre-specified from exploratory	14-19 [Outcome evaluation]
Harms	19	All important harms or unintended effects in each group (for specific guidance see CONSORT for harms)	20 [Process evaluation]
**Discussion**			
Limitations	20	Trial limitations, addressing sources of potential bias, imprecision, and, if relevant, multiplicity of analyses	22-24 [Strengths and limitations, Methodological considerations]
Generalisability	21	Generalisability (external validity, applicability) of the trial findings	23 [Strengths and limitations]
Interpretation	22	Interpretation consistent with results, balancing benefits and harms, and considering other relevant evidence	20-22 [Discussion]
**Other information**			
Registration	23	Registration number and name of trial registry	3, 5 [Abstract, Methodology]
Protocol	24	Where the full trial protocol can be accessed, if available	5 [Methodology]
Funding	25	Sources of funding and other support (such as supply of drugs), role of funders	Entered online during submission
